# Effects of sheep slaughter age on myogenic characteristics in skeletal muscle satellite cells

**DOI:** 10.5713/ab.21.0193

**Published:** 2022-01-03

**Authors:** Yunfei Han, Wenrui Guo, Rina Su, Yanni Zhang, Le Yang, Gerelt Borjigin, Yan Duan

**Affiliations:** 1 College of Food Science and Engineering, Inner Mongolia Agricultural University, Hohhot 010018, China; 2 College of Veterinary Medicine, Inner Mongolia Agricultural University, Hohhot 010018, China; 3 Inner Mongolia Vocational college of Chemical Engineering, Hohhot 010018, China

**Keywords:** Meat Quality, Myofiber Type, Myogenic Regulatory Factors, Myosin Heavy Chains, Wurank Sheep

## Abstract

**Objective:**

The objective of this study was to investigate the effects of sheep slaughter age on myogenic characteristics in skeletal muscle satellite cells (SMSCs).

**Methods:**

Primary SMSCs were isolated from hind leg biceps femoris muscles of Wurank lambs (slaughtered at three months, Mth-3) and adults (slaughtered at fifteen months, Mth-15). SMSCs were selected by morphological observation and fluorescence staining. Myogenic regulatory factors (MRF) and myosin heavy chain (MyHC) expressions of SMSCs were analyzed on days 1, 3, 4, and 5.

**Results:**

The expressions of myogenic factor 5 (Myf5), myogenic differentiation (MyoD), Myf6, and myogenin (MyoG) in Mth-15 were significantly higher in Mth-15 than in Mth-3 on days 1, 3, and 4 (p<0.05). However, MyoG expression in Mth-15 was significantly lower than in Mth-3 on day 5 (p<0.05). The expressions of MyHC I, MyHC IIa, and MyHC IIx in Mth-15 were significantly higher than in Mth-3 on days 1 and 3 (p<0.05), and MyHC IIb were significantly lower than in Mth-3 on days 3 and 4 (p<0.05). In contrast, the expression of MyHC IIx in Mth-15 was significantly lower and MyHC IIb was significantly higher than in Mth-3 on days 5 (p<0.05).

**Conclusion:**

The slaughter age altered the expression of MRFs and MyHCs in SMSCs while differentiation, which caused the variation of myogenic characteristics, and thus may affect the meat quality of Wurank sheep.

## INTRODUCTION

Muscle is a heterogeneous tissue that consists of a large variety of physiologically and biochemically diverse fiber types [1]. According to the structure, contractile properties, metabolic and morphological traits, and skeletal muscle fibers of adult livestock are classified into four types: type I, type IIa, type IIb, and type IIx [2]. Type I and IIa myofibers are predominantly oxidative, use lipids as their main energy source, and perform sustained contractions, whereas type IIb myofibers use glycogen as their primary energy source and perform quick and strong contractions. Type IIx myofibers represent metabolically intermediate types that lie between type IIa and IIb myofibers [3]. The muscle fiber type composition has a profound influence on the biochemical properties of muscle, and meat quality traits of livestock [4,5]. When the proportion of type I and IIa myofibers is higher, the intramuscular fat content is also higher, and the meat’s tenderness, water retention capacity, flavor, juiciness, and other qualities are better [6,7]. In addition, type I and IIa (oxidized) myofibers are rich in myoglobin and hemoglobin; when the proportion of oxidized myofibers is higher, the muscle color is better [8]. Muscle fiber type composition is commonly used to predict meat quality traits. Myosin heavy chain (MyHC) is the most abundant muscle structural protein, comprising about 35% of the protein pool [3].

Four MyHC isoforms (I, IIa, IIx, and IIb) have been identified in the skeletal muscle of representative mammalians and have become a molecular marker for distinguishing myofiber types [2]. Increasing the proportion of MyHC I and IIa expressions can effectively improve meat quality. The proportion of oxidized myofibers can be increased by the addition of nutrients to animal diets [9]. There is much research investigating the modification of skeletal muscle satellite cell (SMSC) proliferation and differentiation to increase the formation of myofibers, to improve the quality of skeletal muscles [10].

Muscle satellite cells are located between the basal lamina and sarcolemma of the muscle. Mauro [11] observed that satellite cells are related to muscle production first. Later, Tsukamoto et al [12] found that muscle satellite cells are the precursor cells of myofibers. When activated, muscle satellite cells proliferate, differentiate into mature muscle cells, and fuse into neighboring cells, thereby affecting hypertrophy of myofibers [13]. The animal’s muscle mass postnatally is changed by the proliferation and myogenic differentiation (MyoD) of SMSC; skeletal muscle development after satellite cell activation is dependent on four myogenic regulatory factors (MRFs), Myf5, MyoD, Myf6 (MRF4), and myogenin (MyoG) [14]. It is widely accepted that Myf5 and MyoD are early factors in satellite cell myogenesis and determine whether muscle satellite cells can be activated to become myoblasts with myogenic properties. Much evidence supports that MyoG and Myf6 regulate myoblast differentiation and fusion myotubes to form myofibers [15,16]. It has been suggested that the muscles of 1+ and 2+-year-old trout have high Myf5 expressions, meanwhile, the expression of MyHC is high, so Myf5 may affect the expression of MyHC [17]. Pin and Konieczny found MRF4 to be expressed mainly in fast-type muscle fibers [18]. Ekmark et al [19] also found that fast-type muscle fibers have higher expression levels of MyoD and lower expression levels of MyoG than slow-type muscle fibers. A further 10 h low-frequency electrical stimulation increases the phosphorylation level of MyoD by 4 times; the content of fast-type muscle fibers increased from 50% to 85% in mice and from 13% to 62% in rats [19]. These studies all demonstrated that muscle fiber types were affected by MRFs, but the specific relationship between MRFs and MyHCs has not been determined.

Some consumers prefer adult sheep meat while others prefer more tender lamb meat. Studies have shown that the main reason for this difference in tenderness is the difference in muscle fiber types [20]. Currently, the process of muscle satellite cell proliferation and differentiation, and the underlying mechanism of MRF regulation of myofiber type transformation is understood. The metabolisms *in vivo* are more complicated and exploring the transformation mechanism is affected by many factors, including exercise, forage, temperature, and growth performance. First, it is more feasible to explore the mechanism at the cellular level. Wurank sheep is a specialty of Abaga Banner of Xilin Gol League in Inner Mongolia Autonomous Region of China, which is an excellent breed formed by natural selection and artificial selection under the ecological conditions of high cold, windy, and arid. By culturing Wurank sheep skeletal muscle cells *in vitro* and testing the expressions of MRFs and MyHCs of SMSCs, the objective of our study was to assess the effects of slaughter age (3-month-old; 15-month-old) of sheep on myogenic characteristics in SMSCs, which may contribute to the development of a preferable muscle fiber type and thus improve meat quality.

## MATERIALS AND METHODS

### Animals and sample collection

Three pairs of twin Wurank sheep from Xilin Gol League, Inner Mongolia, China fed pasture forage and provided free access to water were randomly assigned to one of two groups: 3 slaughtered at three months as lambs (Mth-3), and 3 slaughtered at fifteen months as adults (Mth-15). After slaughter, the biceps femoris muscles samples were collected from hind legs and immediately rinsed and soaked in Hank’s solution, then brought back to the laboratory on ice, and approximately 2 g was taken in the middle of the biceps femoris muscles samples for cell isolation experiment. The study was in compliance with the regulations of Laboratory Animal Welfare and Ethics Committee of Inner Mongolia Agricultural University, China.

### Isolation and culture procedures of muscle satellite cells

The isolation and purification of SMSCs were performed according to the method of Freshney [21] with slight modifications. A tissue block adhesion method was used to separate skeletal muscle cells. The muscle tissues were soaked in 75% ethanol for 30 s under aseptic conditions. After washing with phosphate-buffered saline (PBS), 1 mm^3^ of muscle tissue without fascia and adipose tissue was excised. After 3 washes with PBS and 1 min in a quiescent condition, the supernatant was discarded, and the tissue was placed at certain intervals in culture flasks. A nutrient solution was then added to cell culture flasks and incubated upside down at 37°C and 5% CO_2_ for 2 h. The culture flasks were slowly inverted to ensure that the tissues were covered with the nutrient. When the cells grew by about 60%, the sample block was discarded, and the cells were allowed to continue growing. When they grew to 80%, they were purified, extracted, cultured, and passaged as described below.

Purification and passage: A differential adhesion method was used to purify SMSCs. The separated cells were cultured at 37°C and 5% CO_2_ for 2 hours, the adherent cells were mainly fibroblasts, while the SMSCs will remain in the supernatant. Then the supernatant was transferred to new culture flasks, repeated 2 to 3 times. When the purified cells grew to more than 80%, the nutrient solution was discarded, the culture flask was rinsed 2 to 3 times with PBS, 1 mL of 0.25% trypsin digestion solution was added, and the changes in cell morphology were observed microscopically. When the cell cytoplasm had just shrunk and rounded, the petri dish was gently tapped. When most of the cells dislodged, 3mL of digestion solution was added and the cells were centrifuged at 1,500 r/min for 5 min. The supernatant was discarded, 3 mL of the nutrient solution was added, mixed and to a new flask for 3 to 5 days of cultivation.

### Immunofluorescence staining

In addition to cell morphology observations, we also performed fluorescent staining of SMSC marker genes (paired box 7 [*Pax7*], *MyoD*), and according to the method of Su et al [22] with slight modifications. Immunofluorescence staining was performed on fixed cultured cells. Briefly, fixed cultured cells were permeabilized with 0.3% Triton/PBS, followed by blocking with 3% bovine serum albumin/PBS before incubating with the primary antibodies for Pax7, MyOD, 1:100, at 4°C. The following day, samples were rinsed with PBS and then incubated with the secondary antibodies goat anti-mouse immunoglobulin G (IgG) or goat anti-rabbit IgG. After washing three times with PBS, sections were briefly dried and observed microscopically at ×200 magnification.

### Quantitative real-time polymerase chain reaction

The total RNA of the fifth passage SMSCs was extracted with the Promega EastpSuper total RNA extraction kit (Promega, Beijing, China). The absorbance value of optical density 260 (OD_260_)/OD_280_ was measured with a nucleic acid protein analyzer to detect RNA quality. RNA samples were reverse transcribed into cDNA using GoScript Reverse Transcription Kit (Shanghai Promeg, China). Primer 5.0 was used to design primers (Table 1), and primers were synthesized by Baoshengwu Co., Ltd. (Da Lian, China). Using cDNA as a template, following the instructions of the GoTaq qPCR Master Mix kit (GoScript, Shanghai Promeg, China), the polymerase chain reaction (PCR) solution was prepared for real-time fluorescent quantitative PCR. The glyceraldehyde-3-phosphate dehydrogenase (*GAPDH*) gene was chosen as a reference gene. Relative quantification, or fold of change was quantified as follows:


$$\begin{array}{l} \Delta \Delta Ct=({Ct}_{target}-{Ct}_{reference})\;\text{experiment}-({Ct}_{sample}-{Ct}_{reference})\;\text{control}\\ \text{Fold}\;\text{of}\;\text{change}=2^{-\Delta \Delta Ct} \end{array}$$

### Statistical analysis

Statistical analysis was performed with SPSS 18. The normality of data distribution and homogeneity of variance was tested with the Shapiro-Wilk test and the Levene test, respectively. Data were subjected to one-way analysis of variance (ANOVA) with experimental groups (Mth-3 vs Mth-15) or culture times (days 1, 3, 4, and 5) as the fixed factor. If the ANOVA assumptions were violated, a nonparametric test or a Welch correction was applied when appropriate. The results are shown as relative expressions and error bars in the figures. Differences among means with p<0.05 were accepted as representing statistically significant differences. Pearson correlation coefficients between MRFs and MyHCs expression in SMSCs of Wurank sheep were calculated to test the strength of the relationship.

## RESULTS AND DISCUSSION

### Effect of sheep slaughter age on MRF expression in SMSCs

**Figure 1:**
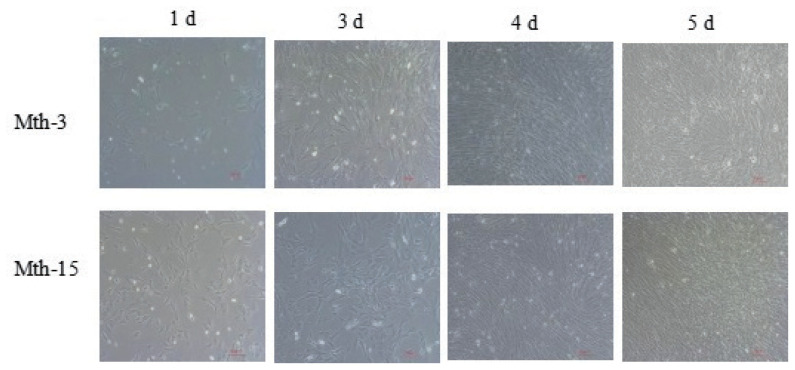
Morphology of Wurank Sheep skeletal muscle satellite cells (10×10). The cell morphology of Mth-3 (slaughtered at three months) and Mth-15 (slaughtered at fifteen months) at different culture stages for days 1, 3, 4, and 5.

**Figure 2:**
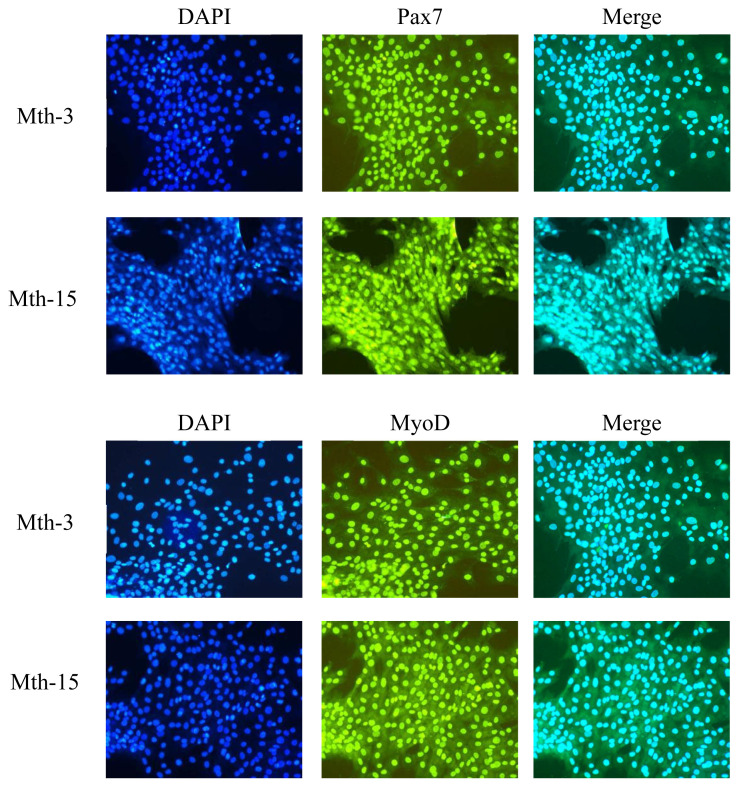
Immunofluorescence analysis of skeletal muscle satellite cells of Mth-3 (slaughtered at three months) and Mth-15 (slaughtered at fifteen months) (10×20). The nuclei of cells were stained with DAPI displayed in the first column. Cells expressed Pax7 and MyoD were shown in the second column, and the merged images were shown in the last column. DAPI, 4′, 6-diamidino-2-phenylindole; Pax7, paired box 7; MyoD, myogenic differentiation.

**Figure 3:**
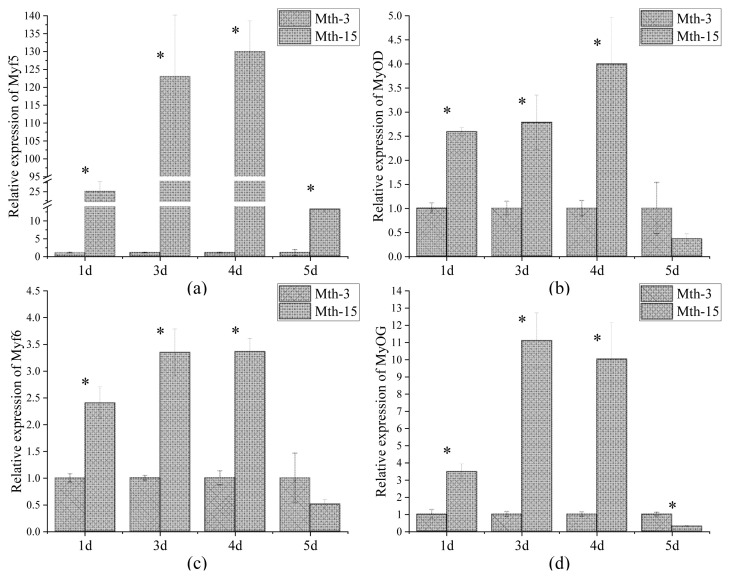
*MRF* gene relative expression in skeletal muscle satellite cells of Wurank sheep. *MRF*, myogenic regulatory factors. * Indicates that there is a significant difference in gene expression of sheep between Mth-3 (slaughtered at three months) and Mth-15 (slaughtered at fifteen months) (p<0.05).

Skeletal muscle satellite cells were isolated by tissue block separation from Wurank sheep slaughtered at 3 or 15 months of age to study their myogenic characteristics and the effect of MRFs on myofiber types during cell proliferation and differentiation. The morphology of SMSCs in the two groups was consistent with the known differentiation processes of SMSCs [23,24]. Pax7 and MyoD immunofluorescence staining of SMSCs cultured *in vitro* were positive (Figure 2). MRFs are the key inducers of skeletal muscle growth, to study the effect of slaughter age on the differentiation of skeletal muscle cells, we observed the expression of MRFs during cell proliferation and differentiation. Myf5 is the first MRF to be expressed in activated satellite cells and MyoD is sequentially expressed in the newly formed fibers. On days 1, 3, and 4 of cell culture, the relative expressions of MyoD and Myf5 in Mth-15 were significantly higher than that of Mth-3 (p<0.05). Myf5 expression was extremely low in Mth-3 during the cultivation process (Figures 3a and 3b). Previous studies demonstrated that Myf5 and MyoD can complement each other in function [16,25], therefore, we speculated that the differentiation of SMSCs in Mth-3 was mainly regulated by MyoD, whereas in Mth-15, the expressions of Myf5 and MyoD were high and thus both may regulate cell differentiation. On day 5, the expression of Myf5 in Mth-15 was significantly higher than in Mth-3 (p<0.05). Moreover, MyoD expression in Mth-15 was lower than that in Mth-3 (p>0.05) (Figure 3a, 3b). MyoG and Myf6 are mainly involved in the differentiation and fusion of myotubes in the late stage, affecting muscle hypertrophy [26]. On days 1, 3, and 4 of SMSCs cultured, Myf6 and MyoG expressions in Mth-15 were significantly higher than those of Mth-3 (p<0.05); On day 5, the expressions of Myf6 and MyoG in Mth-15 were lower than those in Mth-3 (Figure 3c, 3d), indicating that myotubes develop and fuse faster in Mth-15, agreeing with the observations on their cell morphology. Moreover, the expression of MyoG and Myf6 followed same trend during the culture of SMSCs. Myf6 and MyoG have high homology, which promotes each other and jointly regulate muscle differentiation. From the expressions of MRFs combined with the morphological observations of cell culture, our findings that in days 1 to 3 of cell culture, when there is a rapid growth stage of SMSCs, the expressions of MRFs also peaked on days 3 (Figures 1, 5). Wang et al [27] suggested that the *in vitro* expression of MyoD and MyoG in sheep SMSCs reached the maximum on day 2, and then decreased, which is consistent with our observations. MRFs expressions differ in MSCSs of Mth-3 and Mth-15, and the expressions of MRFs promote the proliferation and differentiation for satellite cells.

### Effect of sheep slaughter age on MyHCs expression in SMSCs

**Figure 4:**
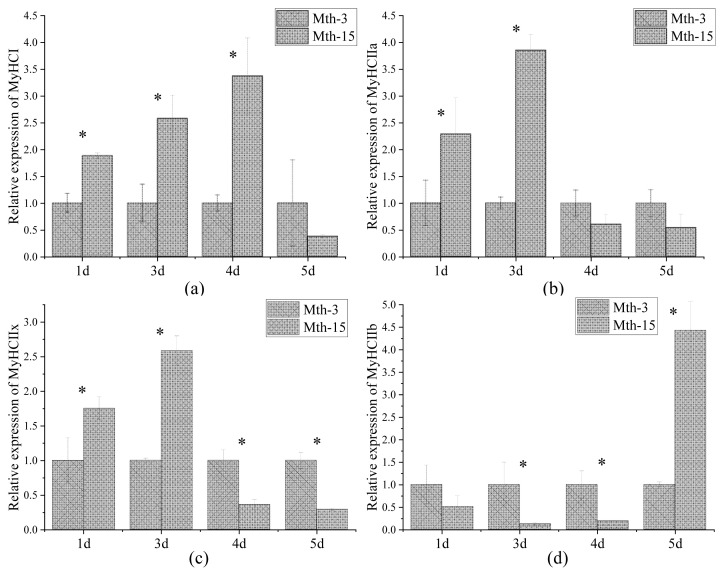
*MyHC* gene relative expression in Wurank sheep skeletal muscle satellite cells. *MyHC*, myosin heavy chain. * Indicates that there is a significant difference in gene expression of sheep between Mth-3 (slaughtered at three months) and Mth-15 (slaughtered at fifteen months) (p<0.05).

**Figure 5:**
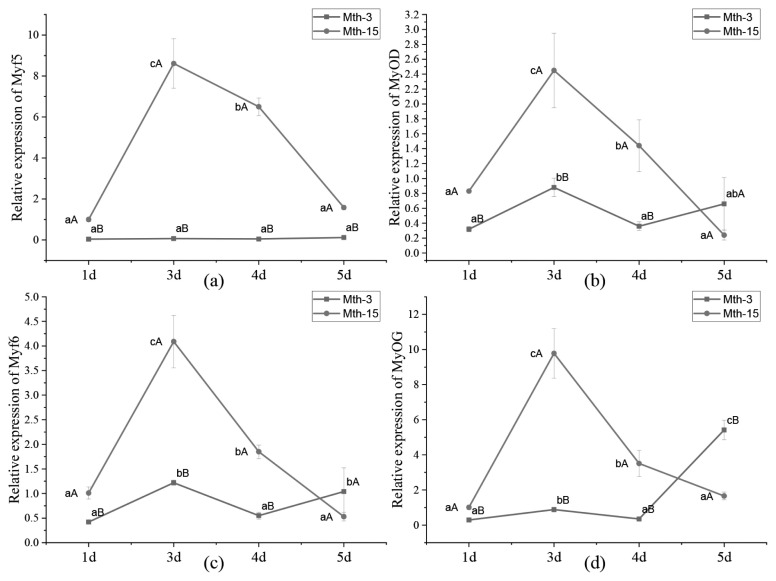
*MRF* gene relative expression in Wurank sheep skeletal muscle satellite cells. *MRF*, Myogenic regulatory factors. ^a–c^ Indicates that there is a significant difference in gene expression of cells during different culture stages (days 1, 3, 4, and 5) (p<0.05). ^A,B^ Indicates that there is a significant difference in gene expression of sheep between Mth-3 (slaughtered at three months) and Mth-15 (slaughtered at fifteen months) (p<0.05).

Skeletal muscle fiber types are distinguished according to the predominantly expressed isoform of MyHCs, which are referred to as type I, IIa, IIx, and IIb. MyHCs have become a molecular marker for distinguishing myofiber types and studying myofiber characteristics [2]. MyHC I expression in Mth-15 was significantly higher than that of Mth-3 (p<0.05) on days 1, 3, and 4, but on day 5 of cell culture, the expression in Mth-15 was lower than that of Mth-3 (Figure 4a). The expression of MyHC IIa in Mth-15 on days 1 and 3 was significantly higher than that of Mth-3 (p<0.05). On days 4 and 5, the expression of MyHC IIa in Mth-15 was lower than that of Mth-3 (Figure 4b). The MyHC IIx expression of SMSCs in Mth-15 on days 1 and 3 was significantly higher than that of Mth-3 (p<0.05). On days 4 and 5, the expression of MyHC IIx in Mth-15 was significantly lower than that of Mth-3 (p<0.05) (Figure 4c). The expression of MyHC IIb in Mth-15 was significantly lower than that in Mth-3 on days 1, 3, and 4, while the expression was significantly higher than that in Mth-3 (p<0.05) on day 5 (Figure 4d). All in all, on days 1 and 3, the expressions of MyHC I, MyHC II, and MyHC IIx in Mth-15 were higher than in Mth-3. Gao et al [28] found that the expression of MyHC I and MyHC IIa decreased with increasing sheep age. After the myotubes formation stages (day 5), the expressions of MyHC I and MyHC IIa in Mth-3 were higher than that in Mth-15, which suggest that the types I and IIa myofibers may be lower in the muscle of Mth-15 than that in Mth-3, which is consistent with a previous study [28]. Previous studies demonstrated the transformation of myofiber types follows the transformation path of I → IIa →IIx → IIb, and the myofibers of many animals have such transformation potential [29]. From days 4 to 5 of cell culture, the expression of MyHC IIb rapidly increased, indicating the SMSCs of Mth-15 had rapidly converted to MyHC IIb with the formation stages of myotubes (day 4 to 5). The expression of MyHCs changed greatly and rapidly during the *in vitro* culture of aged sheep skeletal muscle cells.

### Effect of MRF expression on MyHC expression in SMSCs

**Figure 6:**
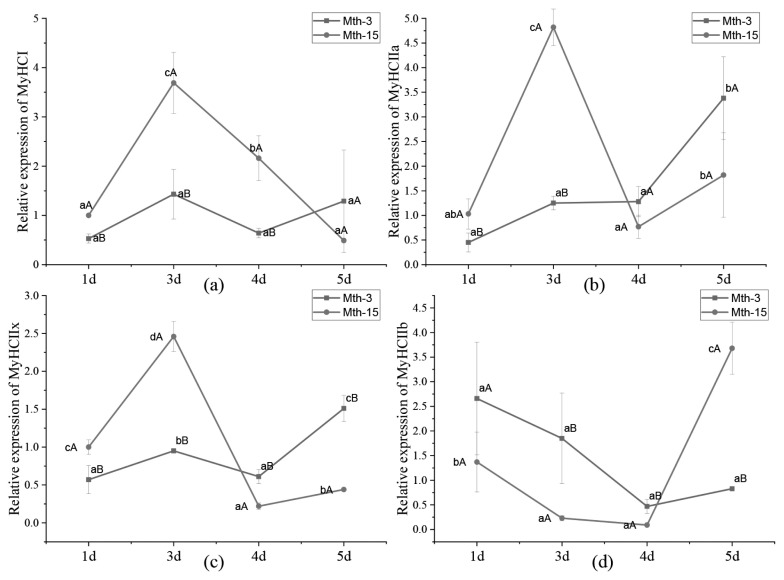
*MyHCs* gene relative expression in Wurank sheep skeletal muscle satellite cells. *MyHCs*, myosin heavy chains. ^a–c^ Indicates that there is a significant difference in gene expression of cells during different culture stages (days 1, 3, 4, and 5) (p<0.05). ^A,B^ Indicates that there is a significant difference in gene expression between Mth-3 (slaughtered at three months) and Mth-15 (slaughtered at fifteen months) (p<0.05).

Churova et al [17] have found that while Myf5 is highly expressed in muscles of 1+ and 2+ years old trout, MyHC expression is also high, therefore, Myf5 may affect the expression of MyHC. In the present study, the correlation matrix between MyHC and MRF expression in SMSCs of Wurank sheep is shown in Table 2. MRFs expressions were positively correlated with the expressions of MyHC I and MyHC IIa but negatively correlated with MyHC IIb. Myf6 and MyoG were positively correlated with MyHC IIx. The results were supported by Ekmark et al [19] and Zhu et al [30]. Ekmark et al [19] research found that 10 h low-frequency electrical stimulation increases the phosphorylation level of MyoD by 4 times, and the content of fast-type muscle fibers increased from 50% to 85% in mice and from 13% to 62% in rats. Zhu et al [30] found that stable MyoG-transfected differentiating C2C12 cells showed higher mRNA expression of MyHC IIx. Given the significant correlation between MRF and MyHC expression, highlights the importance of MRF in terms of MyHC in the myogenesis of SMSCs. In Mth-5, the expressions of Myf5, MyoD, Myf6, MyoG changes were consistent with MyHC I, but opposite to MyHC IIb (Figure 6), which suggests that MRFs were related to the expressions of MyHCs, and further influences the composition of the muscle fiber types.

### The SMSCs from adults induced the muscle type from oxidative fiber to the fast glycolytic ones

The expressions of MyHC I, MyHC IIa, and MyHC IIx in the SMSCs of Mth-15 were higher in differentiation stages (day 3) but lower in formation stages of myotubes (day 5). Moreover, MyHC IIb in Mth-15 demonstrated an uptrend, indicating that the formation of IIb type fibers was further developed, which reason may be the different growth performance of sheep at different slaughter ages.

The type and composition of muscle fibers are important factors affecting meat quality. When the area ratio of glycolytic muscle fibers is large, carcass weight is higher at this stage, and the ATPase activity and glycogen content of type IIb muscle fibers is higher than that of oxidized muscle fibers. The metabolic process dominated by anaerobic glycolysis causes the pH to drop rapidly after slaughter, contributing to an increase in loss of muscle dripping, which doesn’t contribute to forming pale soft exudative meat [24,31]. In addition, MyHC I and MyHC IIa were rich in myoglobin and hemoglobin. When the proportion of oxidized myofibers is higher, the muscle color is better [8]. The *in vitro* results allowed us to speculate that the type of fiber in muscles is affected by the slaughter age. The older the sheep, the higher the expression of MyHC IIb in skeletal muscle during the formation of muscle fibers, so the higher the content of type IIb muscle fibers. Therefore, we speculate that similar results *in vivo* affect the meat quality, further explaining that the muscles in young animals have better tenderness, juiciness, and sensory quality. Thus, as described by Schönfeldt et al [32] the meat of younger Angora goats, Boer goats, and sheep is more tender than that of older animals.

## CONCLUSION

The present study demonstrated that the sheep slaughter age altered MRFs and MyHCs expression in SMSCs. During the differentiation, the MyHC IIb expression of SMCSs from 15-month-old sheep was higher than that in 3-month-old sheep, which may increase the proportion of type IIb fibers in muscle tissue of carcass, and thus explaining the variation of tenderness, color of meat quality. The results of this study provided a theoretical basis for improving meat quality based on the proliferation and differentiation of SMSCs of sheep.

## Figures and Tables

**Table 1:** Primer sequences used for quantitative real-time polymerase chain reaction analysis

Gene	Primer sequences	Size (pb)
*MyoD*	F: GCTCCAGAACCGCAGTAAGTT R: CGGCGACAGCAGCTCCATA	106
*MyoG*	F: GAAGCGCAGACTCAAGAAGG R:TGCAGGCGCTCTATGTACTG	129
*Myf5*	F: AGACGCCTGAAGAAGGTCAA R:TGCCATCAGAGCAACTTGAG	220
*Myf6*	F: GGCCAAGGAAGAGAACATGA R: GGCGACAGGATACCATCACT	144
*MyHC I*	F: ATGTTCCCCAAGGCCACCGACAT R: GCAGCCAGCCTATGATGTTGTAGT	176
*MyHC IIa*	F: CATTGACGTTGATCACACCCAGTAT R: TTGTACTGGATGCAGAAGACAGACT	207
*MyHC IIb*	F: AGGGAAACTGGCTTCTGCTGATATT R: CTTGACTCACGAAGGCATAGTCATAT	182
*MyHC IIx*	F: GGAGGAACAATCCAATGTCAAC R:GTCACTTTTTAGCATTTGGATGAGTTA	106
*GAPDH*	F: GGTCGGAGTGAACGGATTTG R: TGGCAACGATGTCCACTTTG	83

*MyoD*, myogenic differentiation; *MyoG*, myogenin; *Myf5*, myogenic factor; *MyHC*, myosin heavy chain; *GAPDH*, glyceraldehyde 3-phosphate dehydrogenase.

**Table 2:** Correlation matrix between MyHC and MRF expression in SMSCs of Wurank sheep

Item	Myf5	MyOD	Myf6	MyOG
MyHC I	0.890^**^	0.991^**^	0.987^**^	0.938^**^
MyHC IIa	0.503	0.627	0.735^*^	0.766^*^
MyHC IIb	−0.502	−0.63	−0.564	−0.449
MyHC IIx	0.424	0.684	0.762^*^	0.725^*^

MyHC, myosin heavy chain; MRF, myogenic regulatory factors; SMSCs, skeletal muscle satellite cells; Myf5, myogenic factor 5; MyoD, myogenic differentiation; MyoG, myogenin.

* p<0.05, significant correlation;

** p<0.01, extremely significant correlation.
